# Unusual combination of elbow dislocation with a retained intraarticular fragment and trochlear fracture and ipsilateral distal radius fracture in an amateur snowboarder: A case report and review of literatures

**DOI:** 10.1016/j.ijscr.2019.01.039

**Published:** 2019-02-01

**Authors:** Dong Kyu Moon, Sun Chul Hwang, Jun Il Yoo, Jin Sung Park

**Affiliations:** Department of Orthopaedic Surgery and Institute of Health Sciences, Gyeongsang National University School of Medicine and Gyeongsang National University Hospital, Jinju, Republic of Korea

**Keywords:** Elbow, Distal radius, Dislocation, Fracture, Snowboard injury

## Abstract

Introduction: The most common damage caused by snowboarding is wrist injury. However, Elbow injuries are relatively rare. In general, elbow dislocation with ipsilateral distal radius fracture is also very rare.

Presentation of case: We present an 18-year-old right hand dominant male with distal radius extra-articular fracture and elbow fracture dislocation. Computed tomographic scan and Magnetic resonance image of elbow joint showed retained intra-articular fragment, trochlear fracture, and the humeral attachment site of lateral collateral ligament (LCL) with rupture. The ruptured LCL was repaired, and then, the distal radius fracture was fixed with a volar distal radius locking plate. At one year after surgery, the patient did not complain of any subjective symptoms or functional deficit.

Discussion: The injury mechanism would fall into the outstretched arm state, leading first to hyperextension of the wrist, resulting in fracture of the distal radius. The remaining force is then applied to the elbow joint by an external rotation and valgus moment arm, resulting in the rupture of the LCL of the elbow joint. In this state, the remaining force is transmitted through the olecranon to the trochlea as a shear force, resulting in osteochondral fracture and subsequent dislocation of the elbow joint.

Conclusion: Combined injury of extremity from snowboarding is relatively rare, but we experienced a case of elbow dislocation combined with distal radius fracture caused by fall on outstretched hand on the snow surface during snowboarding in a young adult.

## Highlights

• Most common damage caused by snowboarding is wrist injury.
• Mechnism of elbow dislocation resulting from falls on the outstretched hand.
• Elbow dislocation with ipsilateral distal radius fracture is not a common injury.
• Such combined injury caused by fall on outstretched hand on the snow surface during snowboarding.

## 1 Introduction

Most injuries in snowboarding occurred as a result of jumping and losing control and most injuries occurred by fall on the snow surface [[1], [2], [3]]. When falling, injury occurs when snowboarders put their hands on the snow surface with their legs fixed on the snowboard. Therefore, in the amateur snowboarder, the most common damage caused by snowboarding is wrist injury, followed by shoulder injury. However, elbow injuries are relatively rare, ranging from 1.5 to 3% [4]. The most common type of injuries is fractures, followed by contusion and sprains, but dislocations are relatively rare [5]. Extremity injury from snowboarding is mostly single injury, and combined injury is rare unless it is collisional injury. Elbow dislocation with ipsilateral distal radius fracture is not a common injury. Generally, there have been only few cases reported, and there have been no reported cases associated with snowboard injuries [[6], [7], [8], [9]]. We present an unusual case of elbow fracture dislocation with ipsilateral distal radius fracture in young amateur snowboarder and reviewed literatures and discuss the injury mechanism and management. This case is reported in line with the SCARE criteria [10].

## 2 Presentation of case

**Fig. 1:**
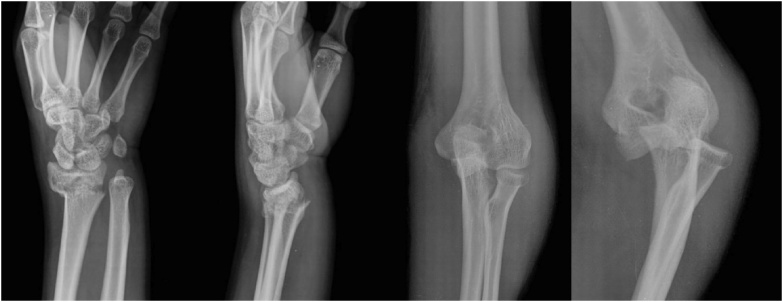
Simple radiopraphs of the left wrist and elbow at the time of injury demonstrate a distal radius fracture and posterior dislocation of the elbow joint.

**Fig. 2:**
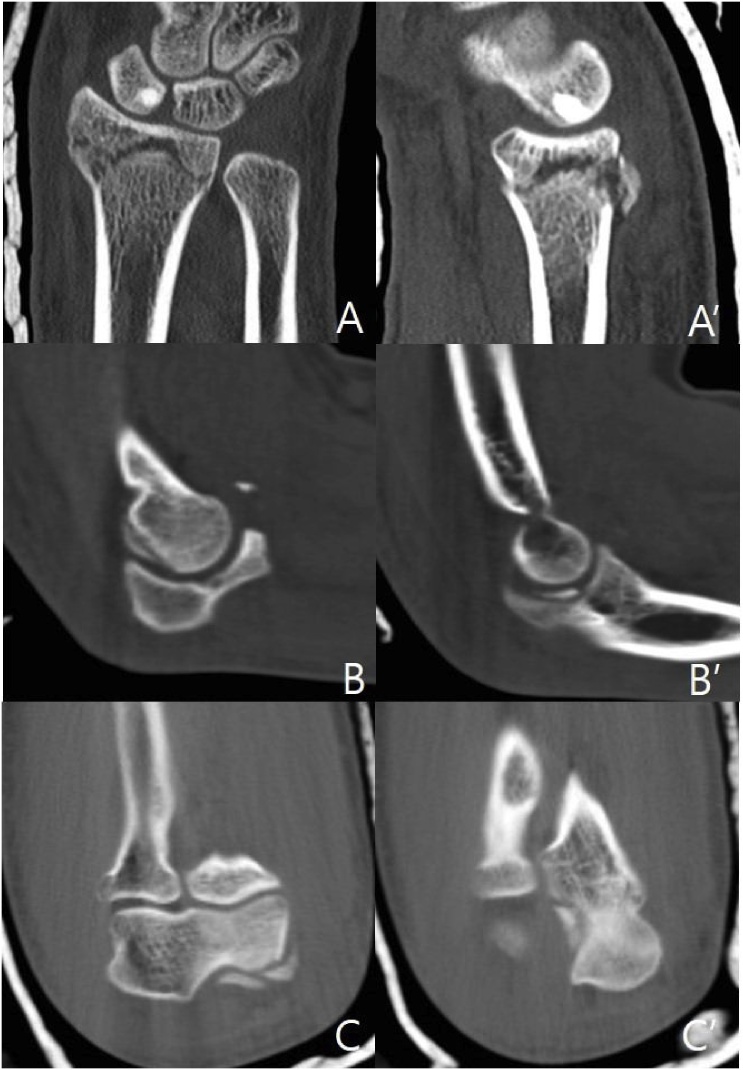
A and A’ CT scan of the left wrist demonstrate AO/OTA classification type A3 distal radius fracture. B and B’ Sagittal CT scan of the left elbow joint demonstrate coronoid tip fracture, trochlea osteochondral fracture, and intraarticular osseous body in elbow joint. C and C’ Axial CT scan of the left elbow joint demonstrate trochlea osteochondral fracture, and intraarticular osseous body in elbow joint.

**Fig. 3:**
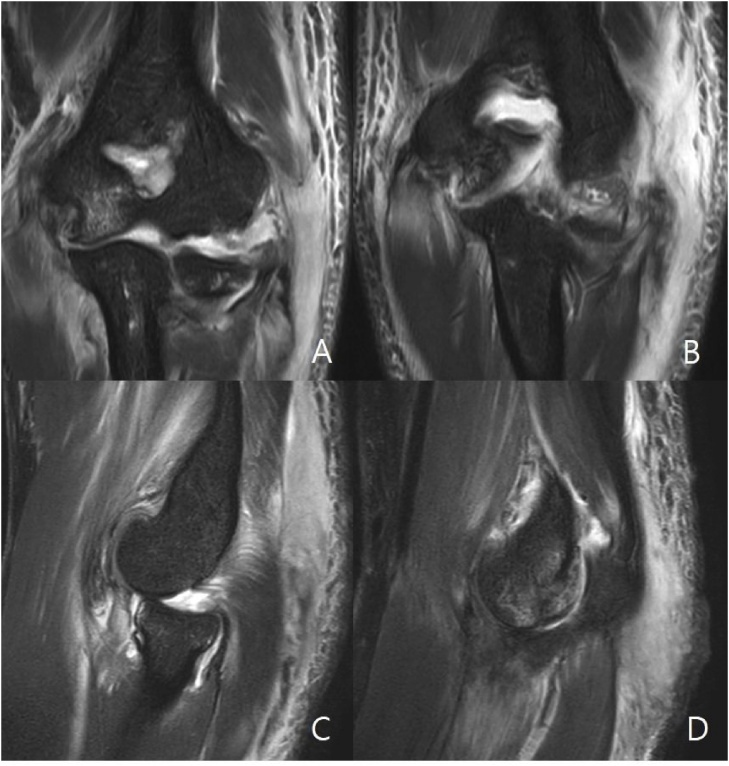
A and B, T2 coronal view of the left elbow demonstrate lateral collateral ligament rupture. C, T2 sagittal view of the left elbow demonstrate posterior subluxation of radial head. D, T2 sagittal view of the left elbow demonstrate trochlear osteochondral fracture and coronoid process tip fracture.

An 18-year-old right hand dominant male visited the emergency room with left elbow pain and ipsilateral wrist pain caused by falling during snowboarding. The patient did not wear protective gear for upper extremity at the time of the injury. He complained of painful swelling in the left elbow and wrist with limited motion. On physical examination, there were not any neurovascular deficit and any wound in his left elbow and wrist. There was posterior dislocation of left elbow joint without definite fracture and extraarticular fracture of distal radius with volar angulation on simple radiographs (Fig. 1). The closed reduction of the elbow joint under sedation was performed gently, followed by reduction of distal radius fracture. CT of elbow joint revealed well reduced ulnohumeral joint with minimally displaced trochlear osteochondral fracture of distal humerus and tip fracture of coronoid process which was less than 25%. However, retention of an intraarticular osseous body from cortex of just beneath radial facet of proximal ulna was found in the ulnohumeral joint (Fig. 2). CT of wrist revealed extraartiuclar fracture with dorsal comminution of distal radius (AO type A3). MRI of elbow joint was performed to evaluate for soft tissue status and other pathology. MRI of the elbow joint revealed high signal change of the humeral attachment site of lateral collateral ligament (LCL) with rupture, high signal change of the common extensor origin, and bone bruise of trochlea. The posterior subluxation of radial head was observed (Fig. 3).

**Fig. 4:**
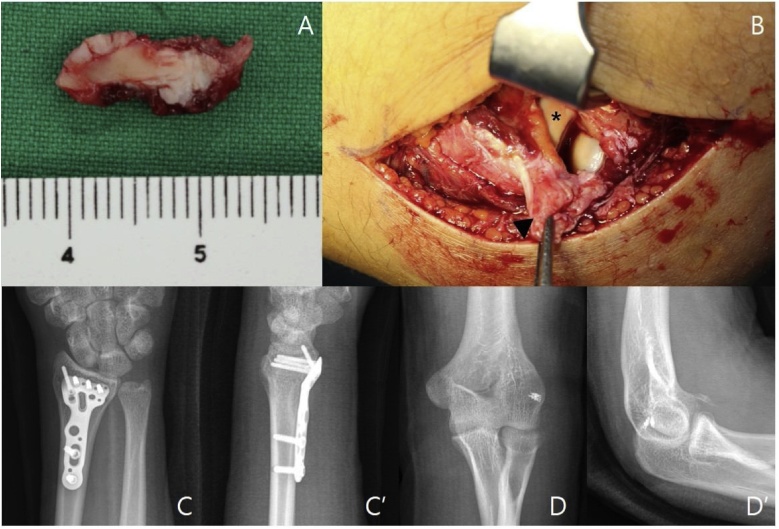
A, Intraoperative gross picture demonstrate intraarticular osseous body. B, Intraoperative gross picture demonstrate complete avulsion of the lateral collateral ligament (LCL) from the humeral attachment site was noticed along with partial avulsion of the common extensor origin and the annular ligament(Arrow head: lateral collateral ligament and common extensor origin, *: Radial head) C and C’, Immediate postoperative simple radiographs of the left wrist. D and D’, Immediate postoperative simple radiographs of the left elbow.

**Fig. 5:**
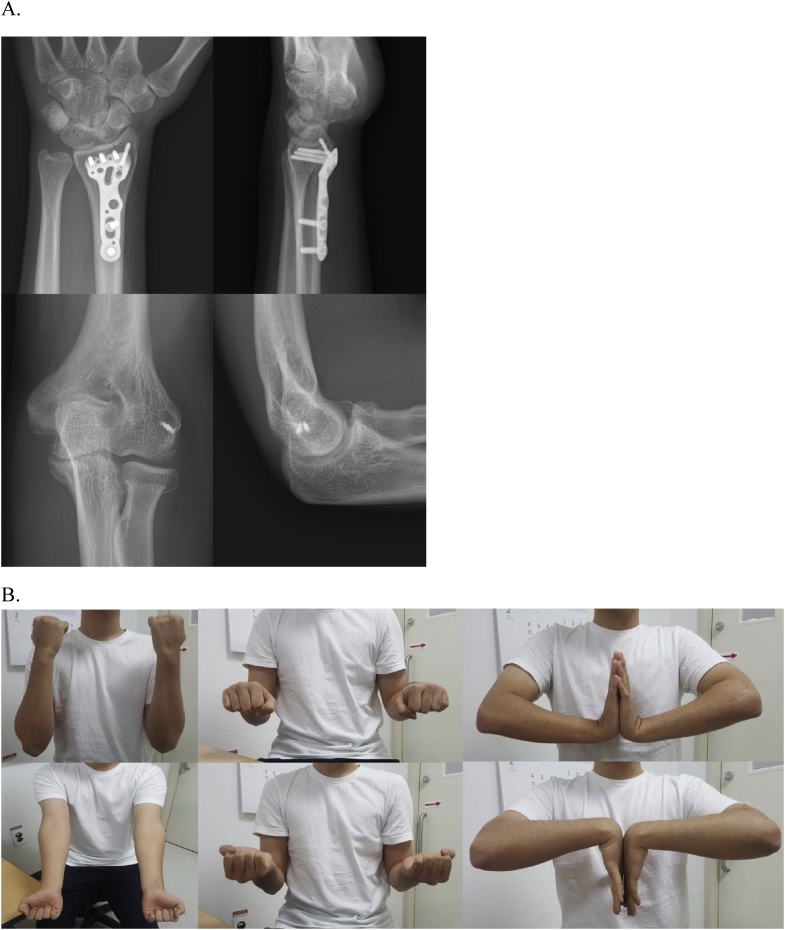
A, simple radiographs of left wrist and elbow at 1 year later after surgery demonstrate complete fracture union of distal radius and congruent elbow joint. B, Clinical pictures at final follow-up demonstrate full range of motion of both wrist and elbow joint.

At five days after injury for control of swelling, surgical treatment was performed. Under the general anesthesia with patient in supine position, the elbow joint was approached through Kocher approach. We found completely avulsed LCL from its humeral attachment site and partial rupture of common extensor origin and the annular ligament during dissection (Fig. 4B). After exposing radiocapitellar joints, we applied valgus stress and widened the joint space to find the intraarticular osseous body, which was removed using a suction device (Fig. 4A). The minimal displaced posteromedial osteochondral fractured fragment of trochlea was stable during elbow motion, so we decided to treat conservatively. The ruptured LCL and the extensor origin were repaired using metal suture anchors (2.4 mm FASTak, Arthrex^®^) and the annular ligament was repaired using non-absorbable suture. The elbow was confirmed to be stable with concentric reduction under fluoroscopy after the repair. And then, the distal radius fracture was fixed through a volar approach using a volar distal radius locking plate and screws (Acu-Loc volar distal radius plate, Acumed^®^). Final test of the left elbow stability during the joint motion was good after fixation of elbow and wrist. The wound was closed by layer by layer and kept closed suction drain. The wrist and elbow were immobilized with a long arm splint for 1 week. At first postoperative week, functional long arm brace was applied with which he was allowed for passive range of motion exercise of wrist and elbow. The ranges of motion of elbow and wrist recovered fully at three month after surgery. At one year after surgery, the patient did not complain of any subjective symptoms or functional deficit. (Fig. 5)

## 3 Discussion

Elbow dislocation is most commonly associated with damage to soft tissue, such as the ligament around the elbow joint, and fracture of the bony structures such as the radial head, coronoid process, and olecranon [11,12]. Rarely, high-energy injuries such as traffic accidents and fall from a height, may be accompanied by ipsilateral wrist and shoulder injuries [13]. The only few cases of elbow dislocation with ipsilateral distal radius fracture have been reported in the literature. Previously reported cases were young adults who fell on their outstretched hands after falling from a height or falling on their bikes while bicycle riding. In elderly, patients fell on their outstretched hands after falling from a chair height [[6], [7], [8], [9]]. However, Extremity injury from snowboarding is mostly single injury, and combined injury is rare unless it is high energy trauma such as collisional injury [5]. Elbow fracture dislocation with ipsilateral distal radius fracture caused by snowboarding has not been previously described in the literature. In our case, this combined injury was caused by fall on snow surface. In previous reports, except for elderly patients, all were associated with relatively high energy trauma. But, this case is relatively low energy trauma, which is different from other cases.

The most common variety of elbow dislocation is posterior [1,2]. The mechanism of posterolateral rotatory posterior displacement of the elbow is responsible for most posterior dislocations resulting from falls on the outstretched hand [14]. Vaishya et al. [6] reported injury mechanism of elbow dislocation and ipsilateral distal radius fracture would be a single-impact theory that resulted in a compressive force that, when directed on the outstretched hand, fractured the distal end of the radius first. Since the energy is enormous, it travels to the hyperextended and valgus elbow, resulting in a posterior dislocation of the elbow joint. Since the force of the fracture is acting towards the radial column, the remaining force is only transmitted along the ulna, thus forcing the ulnar groove out of the trochlea, and thus causing posterior dislocation. In this case, it is assumed that damage was caused by similar mechnism. We hypothesized that the injury mechanism would fall into the outstretched arm state, leading first to hyperextension of the wrist, resulting in fracture of the distal radius. The remaining force is then applied to the elbow joint by an external rotation and valgus moment arm, resulting in the rupture of the LCL of the elbow joint [13]. However, there is a difference in this case that was not seen in other cases, the remaining force is transmitted through the olecranon to the posteromedial aspect of the trochlea as a shear force, resulting in osteochondral fracture and subsequent dislocation of the elbow joint. Such osteochondral fractures of the trochlear posterior aspect are relatively uncommon and require surgery if the osteochondral fracture is severe or unstable [15].

Elbow dislocation often is associated with a fracture of the distal aspect of the capitellum, or radial head, which appear as intraarticular osseous bodies [13]. However, as in our case, there is no report that a fragment near the coronoid process base is interposed within a joint. We could not identify the intraarticular osseous body on simple radiographs and intraarticular osseous body on CT. Therefore, CT scanning after reduction of the elbow dislocation may be useful for finding pathologies that are not identified in simple radiographs.

In elbow dislocation, non-surgical treatment can produce good results in most cases. In general, surgery is indicated for acute elbow dislocations in two situations. The first occurs when the elbow requires flexion beyond approximately 50–60° to remain reduced. The second occurs when elbow dislocation is associated with unstable fractures around the joint; fracture-dislocation [16]. In this case, the dislocated elbow joint was reduced afferently, and the elbow joint was stable. But, a retained intraarticular osseous body was observed in the elbow joint in CT scan, and the fragment removal was necessary to prevent additional articular cartilage damage. The trochlear fracture in the elbow fracture dislocation is usually caused by coronal shear forces, most of which occur in the anterior, accompanied by capitulum fractures [[17], [18], [19]]. In this case, the fracture line is located in the posteromedial portion and is presumably caused by the mechanism described above. Since there was no osteochondral fracture displacement, it was able to be treated conservatively. Successful treatment was achieved through proper immobilization and rehabilitation.

## 4 Conclusion

Combined injury of extremity from snowboarding is relatively rare, but we experienced a case of elbow dislocation combined with distal radius fracture caused by fall on outstretched hand on the snow surface during snowboarding in a young adult. Therefore, careful clinical and radiological examinations are necessary.

## Conflicts of interest

All of the Authors declare that they have no conflict of interest either personally or with any of their relatives.

## Funding

All authors declare that they did not receive any source of funding by any mean to run this case report. They wrote this paper and they edit it on their own fund.

## Ethical approval

The retrospective case report is exempt from ethical approval in our institution.

## Consent

Informed consent was taken from the patient in order to publish this case report.

## Author contribution

Dr. Jin Sung Park: is the corresponding author. He contributed in study design, data collection and analysis, writing paper, and reviewing literature

Dr. Dong Kyu Moon: Study design, data analysis, writing the paper, and reviewing literature.

Dr. Sun Chul Hwang: Study design, data analysis

Dr. Jun Il Yoo: Study design and data analysis

All authors read and approved the final manuscript.

## Registration of research studies

N/A.

## Guarantor

Dr. Jin Sung Park.

## Provenance and peer review

Not commissioned, externally peer-reviewed
